# Effects of Desflurane and Sevoflurane anesthesia on regulatory T cells in patients undergoing living donor kidney transplantation: a randomized intervention trial

**DOI:** 10.1186/s12871-020-01130-7

**Published:** 2020-08-27

**Authors:** Arpa Chutipongtanate, Sasichol Prukviwat, Nutkridta Pongsakul, Supanart Srisala, Nakarin Kamanee, Nuttapon Arpornsujaritkun, Goragoch Gesprasert, Nopporn Apiwattanakul, Suradej Hongeng, Wichai Ittichaikulthol, Vasant Sumethkul, Somchai Chutipongtanate

**Affiliations:** 1 Department of Anesthesiology, Faculty of Medicine Ramathibodi Hospital, Mahidol University, Bangkok, 10400 Thailand; 2 Pediatric Translational Research Unit, Department of Pediatrics, Faculty of Medicine Ramathibodi Hospital, Mahidol University, Bangkok, 10400 Thailand; 3 Research Center, Faculty of Medicine Ramathibodi Hospital, Mahidol University, Bangkok, 10400 Thailand; 4 Vascular and Transplantation Unit, Department of Surgery, Faculty of Medicine Ramathibodi Hospital, Mahidol University, Bangkok, 10400 Thailand; 5 Division of Infectious Disease, Department of Pediatrics, Faculty of Medicine Ramathibodi Hospital, Mahidol University, Bangkok, 10400 Thailand; 6 Division of Hematology and Oncology, Department of Pediatrics, Faculty of Medicine Ramathibodi Hospital, Mahidol University, Bangkok, 10400 Thailand; 7 Division of Nephrology, Department of Medicine, Faculty of Medicine Ramathibodi Hospital, Mahidol University, Bangkok, 10400 Thailand; 8 Department of Clinical Epidemiology and Biostatistics, Faculty of Medicine Ramathibodi Hospital, Mahidol University, Bangkok, 10400 Thailand

**Keywords:** Clinical trial, Inhalation agent, Kidney transplant, Tregs, Volatile anesthesia

## Abstract

**Background:**

Volatile anesthetic agents used during surgery have immunomodulatory effects which could affect postoperative outcomes. Recognizing that regulatory T cells (Tregs) plays crucial roles in transplant tolerance and high peripheral blood Tregs associated with stable kidney graft function, knowing which volatile anesthetic agents can induce peripheral blood Tregs increment would have clinical implications. This study aimed to compare effects of desflurane and sevoflurane anesthesia on peripheral blood Tregs induction in patients undergoing living donor kidney transplantation.

**Methods:**

A prospective, randomized, double-blind trial in living donor kidney transplant recipients was conducted at a single center, tertiary-care, academic university hospital in Thailand during August 2015 – June 2017. Sixty-six patients were assessed for eligibility and 40 patients who fulfilled the study requirement were equally randomized and allocated to desflurane versus sevoflurane anesthesia during transplant surgery. The primary outcome included absolute changes of peripheral blood CD4^+^CD25^+^FoxP3^+^Tregs which measured by flow cytometry and expressed as the percentage of the total population of CD4^+^ T lymphocytes at pre-exposure (0-h) and post-exposure (2-h and 24-h) to anesthetic gas. *P*-value < 0.05 denoted statistical significance.

**Results:**

Demographic data were comparable between groups. No statistical difference of peripheral blood Tregs between desflurane and sevoflurane groups observed at the baseline pre-exposure (3.6 ± 0.4% vs. 3.1 ± 0.4%; *p* = 0.371) and 2-h post-exposure (3.0 ± 0.3% vs. 3.5 ± 0.4%; *p* = 0.319). At 24-h post-exposure, peripheral blood Tregs was significantly higher in desflurane group (5.8 ± 0.5% vs. 4.1 ± 0.3%; *p* = 0.008). Within group analysis showed patients receiving desflurane, but not sevoflurane, had 2.7% increase in peripheral blood Treg over 24-h period (*p* < 0.001).

**Conclusion:**

This study provides the clinical trial-based evidence that desflurane induced peripheral blood Tregs increment after 24-h exposure, which could be beneficial in the context of kidney transplantation. Mechanisms of action and clinical advantages of desflurane anesthesia based on Treg immunomodulation should be investigated in the future.

**Trial registration:**

ClinicalTrials.gov, NCT02559297. Registered 22 September 2015 - retrospectively registered

## Background

Kidney transplantation is the best option for renal replacement therapy in patients with end-stage renal disease (ESRD), often restoring quality of life in ESRD patients. Allograft rejection, an immune-mediated process, is a common cause of transplant failure [1–4]. Evidence indicates CD4^+^CD25^+^FoxP3^+^ cells, commonly known as regulatory T cells (Tregs), play a critical role in preventing graft rejection by suppression of recipient alloimmune response [5–8]. In healthy subjects, Tregs represent up to 5% of peripheral CD4^+^ T cells [9–11]. In kidney transplant patients, high peripheral blood Tregs were associated with stable graft function. Low peripheral blood Tregs was associated with allograft rejection [12–17].

Currently, adoptive transfer of ex vivo expanded Tregs is a promising strategy to induce transplant tolerance and control graft rejection in kidney transplant recipients [18, 19]. It has been investigated for safety and feasibility in phase I trials, i.e., the ONE (NCT02091232) and TRACT (NCT02145325). Identifying Treg-friendly agents from pharmacologic choices in multiple steps of kidney transplant management may also offer an attractive therapeutic strategy [19]. Characterization of Tregs under various treatment conditions may help refine current preventive measures or identify novel therapeutic targets.

Volatile anesthetic agents are widely used for general anesthesia during kidney transplantation. A growing body of evidence from ex vivo and clinical studies [20–26] suggest desflurane and sevoflurane (halogenated ether inhaled agents) exhibit immunomodulatory effects (e.g., cell proliferation, activation, migration, cytokine production) on neutrophils, macrophages, natural killer cells, B and T lymphocytes. These effects may be mediated via activation of volatile anesthetic receptors (i.e., γ-aminobutyric acid type A receptor, nicotinic acetylcholine receptor, serotonin receptor and non-canonical β2-integrins) or via binding to surface adhesion molecules such as integrin leukocyte function associated antigen-1, which express differentially on peripheral blood leukocytes [25, 26]. However, effects of desflurane and sevoflurane on Treg immunomodulation is surprisingly overlooked and has only rarely been investigated. A better understanding of these effects would have translational potential. For example, the early Treg immunomodulation by anesthetic agents may help mitigating the initiation of alloimmune responses during the 24-h perioperative period, and works in conjunction with the standard immunosuppressive regimen to seamlessly maintain the graft survival in LDKT patients.

This interventional trial aims to compare the immunomodulatory effects of desflurane and sevoflurane anesthesia on peripheral blood Treg induction in patients undergoing living donor kidney transplantation (LDKT). Several plasma cytokines were measured as the surrogate outcomes of the volatile anesthetic effects on anti- and pro-inflammatory responses. Evidence from this study would support future investigation of volatile anesthetic agents as part of perioperative management with an aim to improve transplant outcomes.

## Methods

### Trial design and patient enrollment

This prospective, double-blind, randomized intervention trial was approved by the Ethical Clearance Committee on Human Rights Related to Research Involving Human Subjects, Faculty of Medicine Ramathibodi Hospital, Mahidol University (protocol ID 045823) and the protocol was registered to ClinicalTrials.gov (identifier NCT02559297) on September 22, 2015. Patients aged ≥18 years old who received their first living donor kidney transplantation at Ramathibodi Hospital were included in the study. Patients were excluded for hyperacute graft rejection, currently on immunosuppressive drugs due to underlying diseases, receiving blood products during 24-h perioperative period, or patient refusal to participate in the study at any time point. Informed consent was obtained from all subjects. No interim analysis was performed during the trial. This study followed the CONSORT reporting guideline [27].

### Randomization

Randomization was generated in a 1:1 allocation with a block size of 8 and the random number was put in a sealed envelope. Patients were randomly assigned to either desflurane or sevoflurane intervention by drawing a sealed envelope. Randomization took place on the day of surgery just prior to initiation of anesthesia.

### Blinding

Subjects and outcome assessors (including laboratory technicians and all investigators except the designated research coordinator) were blinded to group allocation throughout surgery, laboratory investigation and data collection. Blinding was uncovered at the time of data analysis.

### Interventions

Patients were randomly assigned to receive desflurane or sevoflurane for the maintenance phase of anesthesia. In addition to the randomized inhalation agents, patients received the same regimen of 1–2 mg of midazolam for premedication and intravenous anesthetic agents including 1–2 mcg kg^− 1^ of fentanyl, 1–2 mg kg ^− 1^ of propofol and 0.5–0.6 mg kg ^− 1^ of atracurium for induction of anesthesia and intubation. A balance anesthesia technique was used for maintenance phase. The inhalation agent (sevoflurane or desflurane) was used in conjunction with 50% nitrous oxide in oxygen. Ventilation was adjusted to maintain normocarbia. End-tidal anesthetic gas monitoring was used to ensure 1.0–1.5 minimum alveolar concentration (MAC) of the inhalation agent during maintenance phase in both groups.

During anesthesia, blood pressure, heart rate, oxygen saturation, ETCO_2_, and temperature were monitored and recorded. Blood pressure was maintained within 20% of baseline values. Hypotension was managed by intravenous fluid and ephedrine IV bolus as needed. Total doses of intravenous medications were recorded. All patients in both interventions were transferred to the kidney transplant unit for postoperative care.

### Blood sample collection

Venipuncture was performed at three time-points; pre-exposure (0-h) and post-exposure (2-h, and 24-h) to inhalation agents. Two tubes of 0.5-ml EDTA blood were collected at each time point, one for Treg enumeration and the other for cytokine measurement.

### Outcome measures

The primary outcome was the absolute change in number of peripheral blood CD4^+^CD25^+^FoxP3^+^Tregs, which was measured by flow cytometry and expressed as the percentage of the total population of CD4^+^ T lymphocytes at pre-exposure (0-h) and post-exposure (2-h and 24-h) to anesthetic gas. A secondary outcome was the plasma level of anti-inflammatory cytokine IL-10 (the major cytokine produced by Tregs), TGF-β1 (anti-inflammatory cytokines produced by many types of cells and required for Tregs differentiation), and pro-inflammatory cytokines produced by T helper (Th) 1/Th2, i.e., GM-CSF, IFN-ɣ, IL-2, IL-4, IL-5, IL-12, IL-13 and TNF-α, which measured by multiplex immunoassay. All measurements were performed in triplicate.

#### Treg enumeration by flow cytometry

Peripheral blood mononuclear cells (PBMC) were isolated by density gradient centrifugation. Approximately 5 × 10^5^ cells were suspended in 20 μL phosphate buffer saline (PBS) in the presence of cell surface marker antibodies (APC-CD4, PE-Cy7-CD25) (#MHCD0404, #25–0259-41; ThermoFisher, Florence, KY), mixed well and incubated at room temperature for 15 min. Thereafter, cells were permeabilized and intracellularly stained using FoxP3-FITC antibody (#11–4776-42; ThermoFisher). Flow cytometry (BD FACSVerse with BD FACSuite software; BD Bioscience, San Jose, CA) was used to measure the number of Tregs, expressed as a percentage of CD4^+^CD25^+^FoxP3^+^ T cells among the CD4^+^ cell population. The estimated number of CD4 + cells and Treg were calculated by determining the ratio of CD4 + cell count and CD4 + CD25 + FoxP3 + cell count, respectively, to the total count in the flow cytometry, and then multiplied by the number of white blood cells measured from the complete blood count (CBC) which ordered at the pre-operative and post-operative evaluations.

#### Cytokine measurement by multiplex immunoassay

Multiplex cytokine immunoassay was performed by BioPlex-200 system (Bio-Rad, Hercules, CA). GM-CSF, IFN-ɣ, IL-2, IL-4, IL-5, IL-10, IL-12, IL13, and TNF-α were detected by BioPlex Pro human cytokine Th1/Th2 assay (Bio-Rad), and TGF-β1 was measured by single-plex custom assay (Bio-Rad) as the manufacturer’s instruction.

### Sample size estimation and statistical analysis

There was no data related to sevoflurane and desflurane anesthesia on Treg immunomodulation available at the initiation of the study. Nevertheless, Pirbudak Cocelli L et al. [21], showed that sevoflurane and desflurane anesthesia caused a significant difference in total lymphocyte count at 2-h post-induction in patients undergoing abdominal surgery. Since Treg is a subset of lymphocytes, our study then adopted mean difference and standard deviation to calculate the effect size. The nQuery Advisor program was applied for sample size calculation. Accordingly, at least 40 patients (20 patients per group) were required to determine statistically significant mean difference between groups (the effect size of 0.915, alpha = 0.05 and power = 80%).

Statistical analysis was performed by Excel and R packages. Data were reported in number, percentage, mean ± SD (or SEM) or median [IQR] as appropriate. Parametric and non-parametric tests were used, as appropriate, to determine difference between groups. ANOVA with Tukey *post-hoc* test was performed for multiple comparison. *P*-value < 0.05 was considered to be statistically significant.

## Results

### Baseline characteristics

**Fig. 1:**
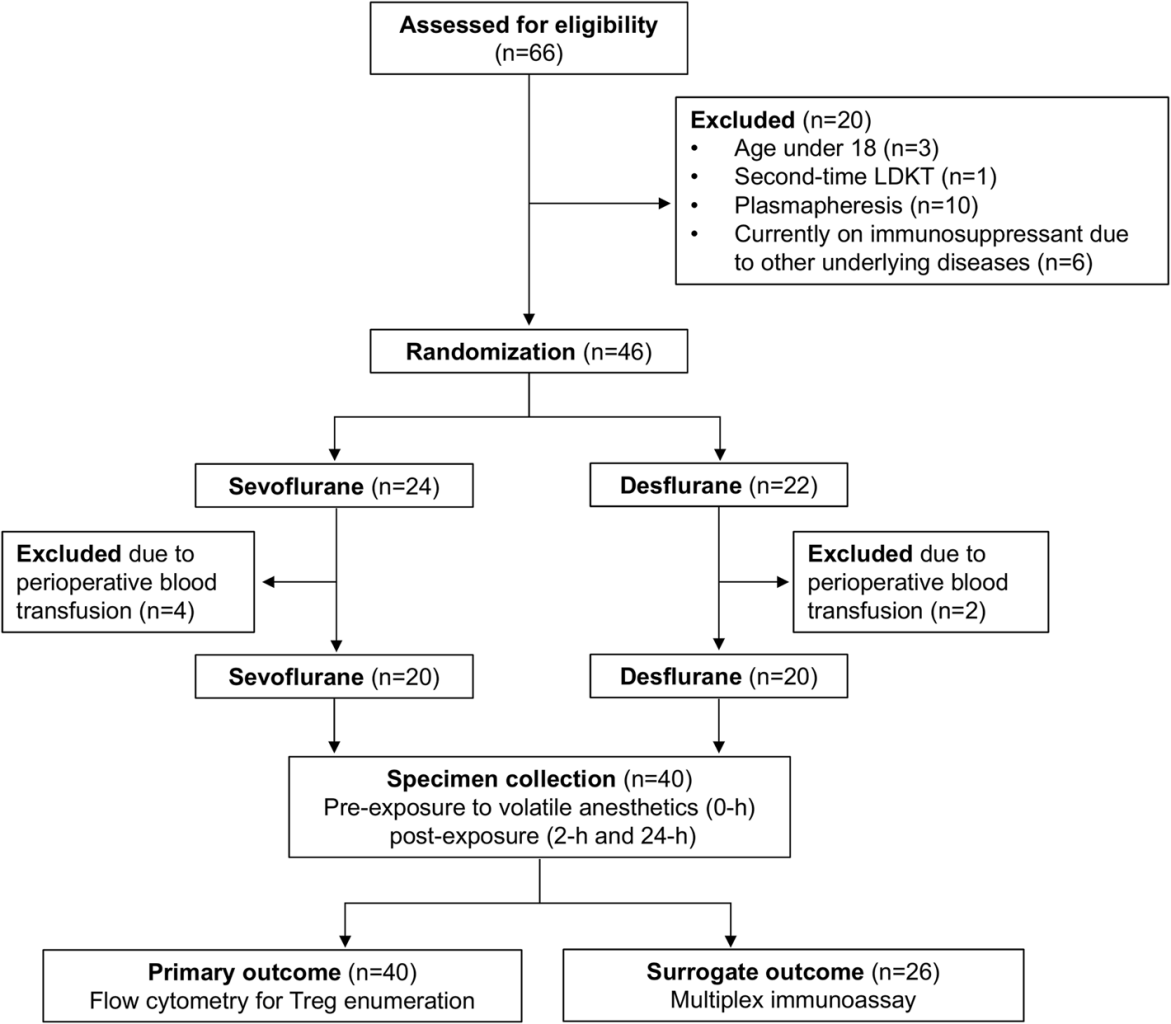
Flow diagram of study participants

Figure 1 shows the flow of the participants in this study. A total of 66 patients were assessed for eligibility and 46 patients who met inclusion criteria were recruited during August 10, 2015 to June 3, 2017 for randomization and allocation to the intervention. Six patients who met exclusion criteria after allocation due to receiving perioperative blood products were excluded. Table 1 shows demographic and clinical data for the 40 patients enrolled in this study. Most variables, including recipient factors, donor factors, protocol immunosuppressive regimens, intraoperative parameters, the dosage of intravenous anesthesia were not significantly different between the intervention groups. Donor age and the estimated blood loss were slightly lower in patients receiving desflurane anesthesia. Factors contributing to ischemic-reperfusion injury, i.e., cold and warm ischemic time, were comparable between groups. Also, anesthesia time was not different between groups (279 ± 42 min vs. 303 ± 45 min, *p* = 0.098). Given that the depth of sevoflurane and desflurane anesthesia was maintained at 1.0–1.5 MAC for each arm, this finding supported that patients were exposed to inhalation agents equally (Table 1).

**Table 1:** Demographic data of LDKT recipients enrolled into the study. If not indicated otherwise: n (%)

Characteristics	Sevoflurane (***n*** = 20)	Desflurane (***n*** = 20)	***P***-value
**Recipient**			
Age (year), mean ± SD	37.6 ± 10.5	42.3 ± 10.6	0.163
Gender, male	13 (65)	15 (75)	0.490
BMI (kg/m^2^), median [IQR]	21.8 [19.2, 24.4]	22.3 [21.5, 25.1]	0.301
Hypertension	18 (90)	19 (95)	0.999
Diabetes mellitus	4 (20)	0 (0)	0.106
ADPKD	1 (5)	0 (0)	0.999
Hemodialysis	18 (90)	15 (75)	0.407
Peritoneal dialysis	2 (10)	5 (25)	0.407
Baseline creatinine (mg/dL), median [IQR]	7.7 [5.4, 11.8]	7.2 [5.2, 12.9]	0.797
Pretransplant PRA (%), median [IQR]	0 [0, 0]	0 [0, 0]	0.999
HLA-A + B + DR mismatch, median [IQR]	1 [1, 1.5]	3 [1, 3]	0.371
White blood cell count (cells/mL), mean ± SD	6130 ± 2157	5838 ± 2072	0.665
Lymphocyte (%), mean ± SD	21.4 ± 6.7	22.8 ± 9.2	0.586
**Donor**			
Age (year), mean ± SD	44.8 ± 12.8	36.1 ± 11.3	0.029
Gender, male	7 (35)	6 (30)	0.736
Donor warm ischemic time (min), median [IQR]	3.0 [2.0, 4.0]	3.0 [2.0, 5.3]	0.519
Cold ischemic time (min), median [IQR]	26.5 [18.0, 31.5]	26.5 [19.5, 40.8]	0.432
**Protocol immunosuppressive drug**			
Tacrolimus	8 (40)	11 (55)	0.342
Cyclosporin A	3 (15)	2 (10)	0.999
Tacrolimus *plus* basiliximab	7 (35)	5 (25)	0.490
Cyclosporin A *plus* basiliximab	2 (10)	2 (10)	0.999
**Intraoperative variable**			
Recipient warm ischemic time (min), median [IQR]	37.5 [31.5, 44.5]	35.5 [30.0, 41.3]	0.647
Estimated blood loss (mL), mean ± SD	348 ± 124	250 ± 129	0.020
Intravenous fluid (mL), mean ± SD	3716 ± 869	3968 ± 979	0.396
Intravenous ephedrine (mg), median [IQR]	0.0 [0.0, 7.5]	0.0 [0.0, 12.8]	0.701
**Intravenous anesthesia**			
Propofol (mg), median	175.0 [127.5,	200.0 [170.0,	0.26
[IQR]	200.0]	200.0]	2
Fentanyl (mcg), median	100.0 [100.0,	100.0 [100.0,	0.76
[IQR]	150.0]	156.3]	7
Morphine (mg), median [IQR]	5.5 [3.0, 7.3]	6.0 [3.0, 10.0]	0.678
Midazolam (mg), median [IQR]	2.0 [0.0, 2.1]	2.0 [0.0, 2.5]	0.650
Atracurium (mg), median [IQR]	90.0 [80.0, 110.0]	92.5 [85.0, 101.3]	0.643
**Anesthesia time (min)**	279 ± 42	303 ± 45	0.098

Abbreviations: *ADPKD* Autosomal dominant polycystic kidney disease, *BMI* Body mass index, *HLA* Human leukocyte antigen, *PRA* Panel reactive antibodies

### Effects of Desflurane and Sevoflurane anesthesia on Tregs in LDKT recipients

**Fig. 2:**
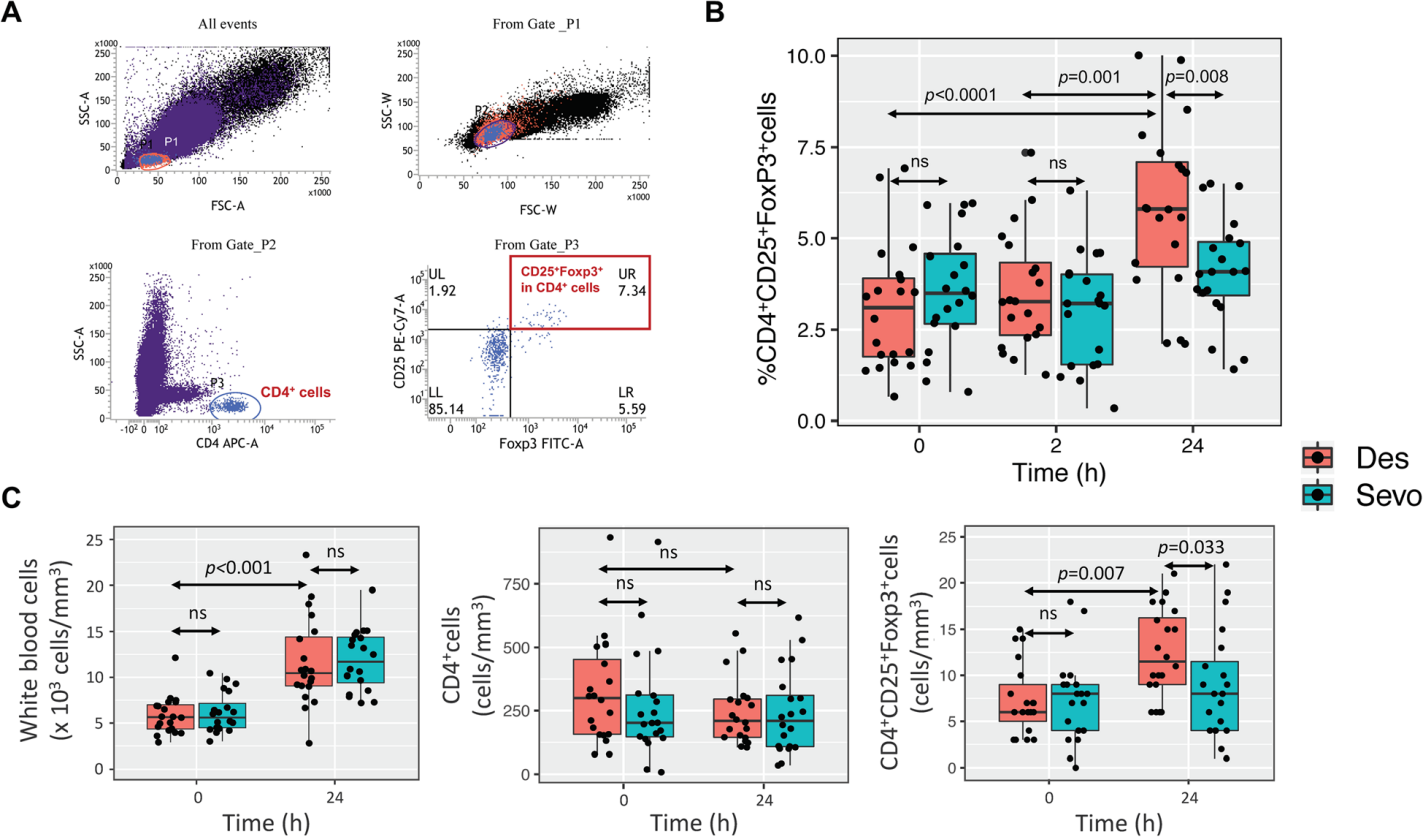
Effects of desflurane and sevoflurane anesthesia on the CD4 + CD25 + FoxP3+ Tregs (*n* = 20 per group). **a** Representative gating strategy of Treg enumeration. **b** The percentage of Treg in the CD4 + cell population. **c** The absolute number of white blood cells (left), and the calculated numbers of CD4 + cells (center) and Treg (right), in peripheral blood (details in the Method section). The result showed that the LDKT patients who received desflurane anesthesia, but not sevoflurane, had significantly increased Tregs in the peripheral blood at 24-h post-exposure. All experiments were performed in triplicate. Des, desflurane; NS, not significant; Sevo, sevoflurane

Figure 2a demonstrates the gating strategy of flow cytometry and Fig. 2b shows the effects of sevoflurane and desflurane anesthesia on CD4^+^CD25^+^FoxP3^+^ Tregs in peripheral blood of LDKT recipients (*n* = 20 per group). No significant difference of peripheral blood Tregs (mean ± SEM) was observed at pre-exposure (3.6 ± 0.4% vs. 3.1 ± 0.4%; *p* = 0.371) and 2-h post-exposure (3.0 ± 0.3% vs 3.5 ± 0.4%; *p* = 0.319) between desflurane and sevoflurane, respectively. However, at 24-h post-exposure, desflurane group had significantly higher peripheral blood Tregs as compared to sevoflurane group (5.8 ± 0.5% vs. 4.1 ± 0.3%; *p* = 0.008) (Fig. 2b). Within-group analysis showed that the patients receiving desflurane had 2.7% increase in Tregs over 24-h period (*p* < 0.001) (Fig. 2b), while this effect was not observed in sevoflurane group.

Although peripheral blood Treg are commonly presented in the literatures as the percentage of CD25^+^FoxP3^+^cells in the CD4^+^cell population, one argument was that the increment in Treg percentage might be corresponding to the global changes of leukocytes or CD4^+^ T cells in response of surgical procedure and postoperative inflammation but not the influence of inhalation agents. To address this issue, the absolute number of white blood cells (as measured by the complete blood count), and the calculated numbers of CD4+ T cells and CD4^+^CD25^+^FoxP3^+^ Tregs (details in the Method section), were shown in Fig. 2c. Postoperative leukocytosis was observed in both groups as expected (Fig. 2c, the left panel), while the number of CD4+ T cells were not significantly changed during 24-h perioperative period (Fig. 2c, the center panel). Consistently, the number of Tregs (median [IQR]) were comparable between groups at the baseline (7 [6, 11] vs. 8 [4, 9] cells/mm^3^; *p* = 0.780) and were significantly higher in desflurane group (12 [9, 16] vs. 8 [4, 12] cells/mm^3^; *p* = 0.033) at 24-h post-exposure (Fig. 2c, the right panel). This finding suggested the effect of desflurane anesthesia on the peripheral blood Treg induction during 24-h postoperative period.

**Fig. 3:**
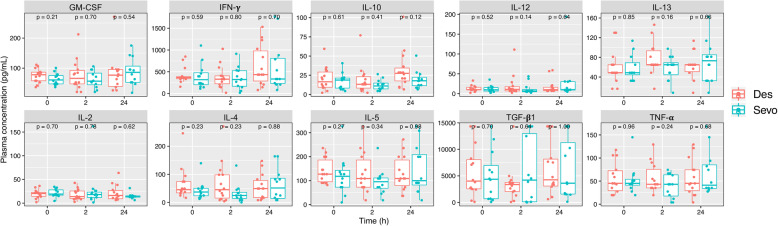
Plasma cytokines were measured by multiplex cytokine immunoassay. Box plots exhibited plasma levels of anti-inflammatory cytokines IL-10 and TGF-β1, and pro-inflammatory cytokines GM-CSF, IFN-γ, IL-2, IL-4, IL-5, IL-12, IL-13 and TNF-α (*n* = 26; 14 desflurane, 12 sevoflurane). IL-10 showed an increased trend over 24-h period in patients receiving desflurane anesthesia. Des, desflurane; Sevo, sevoflurane

Plasma cytokine levels were measured by multiplex immunoassay as a surrogate outcome of immunomodulation possibly influenced by inhalation agents (*n* = 26, 12 sevoflurane and 14 desflurane). Although there was no statistically significant difference between groups in any cytokine, a trend of increased IL-10 was observed in desflurane group as compared to sevoflurane group at 24-h post-exposure (27.5 [17.6, 34.4] vs. 17.8 [11.4, 22.3] pg/mL; *p* = 0.12) (Fig. 3 and Supplementary Table 1). IL-10, the signature anti-inflammatory cytokine produced by Tregs, had an upward trend (1.67-time increased at 24-h as compared to the baseline pre-exposure) in the patients receiving desflurane, whereas other cytokines seemed to be unchanged over the 24-h period (Fig. 3 and Supplementary Table 1). The transient drop of measured cytokines at 2-h was potentially associated with intraoperative factors, e.g., intravenous fluid administration, but not directly influenced by inhalation agents.

**Fig. 4:**
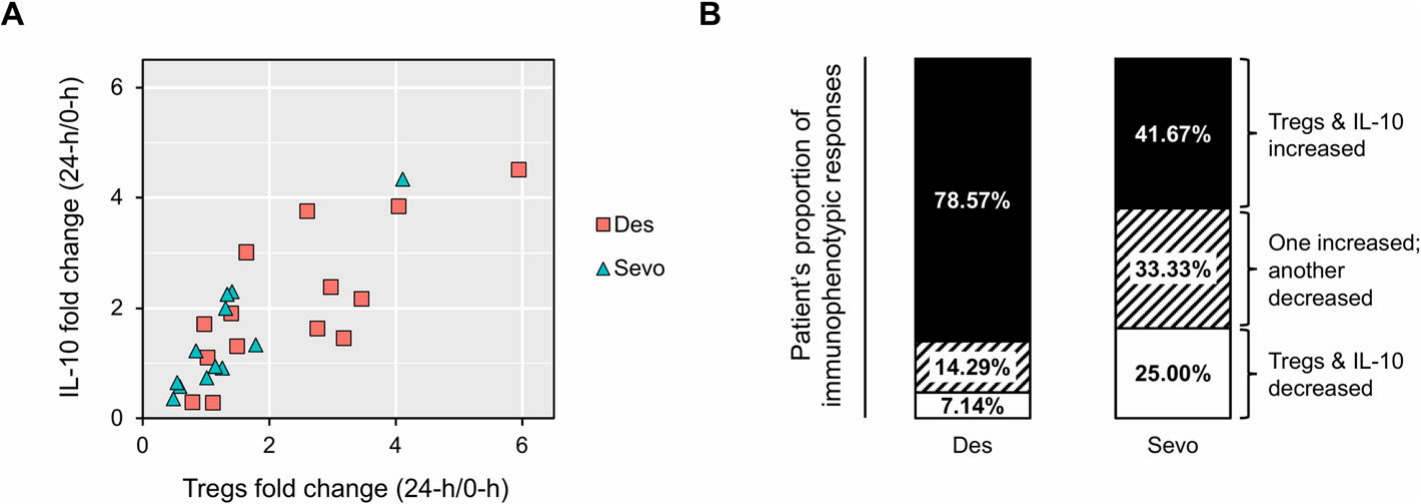
Matched-pair data analysis of Tregs and IL-10 (*n* = 26; 14 desflurane, 12 sevoflurane). Fold change was calculated by Tregs (or IL-10) measured at 24-h divided by that of pre-exposure (0-h) in the same patient, in which fold change > 1 indicated upregulation and fold change < 1 was downregulation. **a** Scatter plot exhibited the positive relationship between Tregs and IL-10 fold changes. **b** Bar plots showed a higher proportion of co-increased Tregs and IL-10 immunophenotypic response in patients receiving desflurane anesthesia. Des, desflurane; Sevo, sevoflurane

An increased trend of plasma IL-10 in the desflurane group was in line with previous results (Fig. 2) and suggested that desflurane anesthesia was associated with IL-10-producing Tregs induction in LDKT patients during the perioperative period. Matched-pair data of Tregs and plasma IL-10 levels in 26 patients (14 desflurane, 12 sevoflurane) were analyzed to observe this immunophenotypic response. Scatter plot showed a positive relationship between Tregs and IL-10 fold changes over 24-h period (Fig. 4a), in which the proportion of patients with increased Tregs and IL-10 immunophenotypic response was higher in the desflurane group (Fig. 4b). Taken together, our findings revealed that desflurane anesthesia induced IL-10-producing Tregs in LDKT recipients over 24-h postoperative period.

## Discussion

Increasing evidence suggests that volatile anesthetic agents exhibit immunomodulatory effects linked to innate and adaptive immunity via induction and suppression of neutrophils, macrophages, NK cells and B and T lymphocytes [25, 26]. However, their effects on Treg have remained unknown. This study, for the first time, showed that desflurane, but not sevoflurane, increased Treg frequency in peripheral blood of LDKT recipients during 24-h perioperative period. Selection of desflurane anesthesia in kidney transplantation may have additional benefits to kidney graft outcome, particularly preventing allograft rejection.

Studies showed that kidney transplant patients who maintained a high level of peripheral blood Tregs were associated with better outcomes [14, 15, 28]. San Segundo D, et al. [14], reported that among 90 kidney transplant recipients, patients who maintained high levels (above 70th percentile) of peripheral blood Tregs at both 6 and 12 months had a better prognosis in the aspect of long-term graft survival after 4 and 5 years follow-up. Liu L, et al. [15], compared peripheral blood Treg levels between 42 patients with stable kidney graft function and ten patients who suffered from chronic rejection. The results showed that Treg levels were significantly higher in the stable group than the chronic rejection group. Alberu J, et al. [28], investigated the association between Treg levels and de novo donor-specific HLA-antibody (DSA) production in 53 kidney transplant patients. Although early development of DSA was not associated with Treg numbers, at 12 months after kidney transplant DSA-negative patients had higher number of peripheral blood Treg.

The mechanisms for which higher peripheral blood Tregs help prevention of allograft rejection and maintenance of transplant tolerance meet the same concept of peripheral regulation in autoimmune reaction [18, 29–32]. On a cellular basis, Tregs utilize four modes of action in peripheral regulation including [29–32]; i) secretion or generation of inhibitory cytokines (e.g., IL-10, IL-35, TGF-β and adenosine); ii) direct killing of targets through Granzyme A/B and perforin-dependent cytolysis; iii) IL-2 consumption through high IL-2R expression which leads to cytokine-mediated deprivation and apoptosis of effector cells; and iv) direct interaction with CTLA-4, LAG-3 and PD1 molecules. Although accumulating evidence would favor contact-dependent mechanisms over non-contact/secretory component alone, these different mechanisms should work in concert to control various immune effector cells and regulate different inflammatory settings [29–32]. Given that breadth of regulatory function on autoimmunity and self-tolerance, changes in peripheral blood Tregs in kidney transplant recipients may shift the balance between allograft rejection and transplant tolerance.

According to these lines of evidence, the increment of Tregs and IL-10 (Figs. 2, 3 and 4) after exposure to desflurane anesthesia should be beneficial to graft outcomes in LDKT recipients. In fact, several drugs routinely used in general anesthesia (besides volatile anesthetic agents) have immunomodulatory properties [25, 26]. It is possible that some of them have positive effects on Tregs. Synergistic effects of multiple Treg-modulated agents may provide a better transplant outcome. Understanding how anesthetic agents exhibit varied effects on the immune system, particularly on Tregs, is important for future development of perioperative medicine in kidney transplantation.

This study was associated with several limitations. First, clinical outcomes, such as short-term and long-term graft survival, that were associated with Treg immunomodulation of desflurane anesthesia were not investigated. Graft survival is influenced by various factors (such as adequacy and toxicity of immunosuppressive drugs, presence of donor-specific antigen, PRA levels, numbers of HLA mismatch). These factors, together with Treg immunomodulation of desflurane, should be taken into account in future studies. Secondly, plasma cytokine levels were measured in 26 out of 40 patients (2/3 of total population in this study) due to the limited budget. A non-significant difference of cytokines between intervention groups may be due to a lack of statistical power, but at least, the upward trend of plasma IL-10 was observed, given the supportive evidence that desflurane anesthesia induced peripheral blood Tregs with potential IL-10 production. Third, the mechanisms of action (MoA) that drive desflurane-Treg immunomodulation were not defined and not the focus of this study. Further studies to characterize receptors and downstream signaling pathways that are responsible for desflurane-Treg effects would give some insight into a new MoA class of volatile anesthetic agent or a new biological process that facilitates transplant tolerance in LDKT recipients.

## Conclusion

In summary, desflurane had the advantage over sevoflurane as the inhalation anesthetic in LDKT patients regarding the increment of peripheral blood Tregs. Further research focused on clinical outcomes and pharmacological actions of desflurane on Treg immunomodulation has translational potential, which could eventually benefit LDKT recipients.

## Supplementary information

**Additional file 1: Table S1.** Effects of sevoflurane and desflurane anesthesia on plasma cytokine levels of LDKT patients.

## Data Availability

The datasets used and/or analyzed during the current study are available from the corresponding author on reasonable request.

## Abbreviations

ADPKD: Autosomal dominant polycystic kidney disease
BMI: Body mass index
ESRD: End-stage renal disease
HLA: Human leukocyte antigen
LDKT: Living donor kidney transplantation
MoA: Mechanisms of action
PRA: Panel reactive antibodies
Th1: T helper 1
Th2: T helper 2
Tregs: Regulatory T cells
