# Supplementary material for: Effects of Desflurane and Sevoflurane anesthesia on regulatory T cells in patients undergoing living donor kidney transplantation: a randomized intervention trial
Source: BMC Anesthesiol. 2020 Aug 27;20:215. doi: 10.1186/s12871-020-01130-7 (PMC7450591; doi:10.1186/s12871-020-01130-7)
Supplement: Supplementary file 1 — Additional file 1: Table S1. Effects of sevoflurane and desflurane anesthesia on plasma cytokine levels of LDKT patients. [file 12871_2020_1130_MOESM1_ESM.docx]

**SUPPLEMENTARY INFORMATION**

**Supplementary table 1**. Effects of sevoflurane and desflurane anaesthesia on plasma cytokine levels of LDKT patients.

| **Variable** | **Sevoflurane, n=12** | **Desflurane, n=14** | ***P-value*** |
| --- | --- | --- | --- |
|  | Median [IQR] | Median [IQR] |  |
| GM-CSF, pg/mL |  |  |  |
| pre-exposure | 60.8 [47.5, 75.4] | 78.1 [58.1, 86.7] | 0.21 |
| 2h post-exposure | 56.8 [44.8, 83.4] | 66.1 [47.5, 92.7] | 0.70 |
| 24h post-exposure | 86.0 [52.8, 106.7] | 76.7 [39.5, 95.4] | 0.54 |
| IFN-gamma, pg/mL |  |  |  |
| pre-exposure | 318.5 [204.8, 479.1] | 365.8 [342.1, 389.4] | 0.59 |
| 2h post-exposure | 322.9 [168.8, 526.4] | 330.8 [223.9, 406.5] | 0.80 |
| 24h post-exposure | 334.2 [231.9, 811.3] | 434.9 [288.5, 980.1] | 0.70 |
| IL-2, pg/mL |  |  |  |
| pre-exposure | 19.2 [16.8, 28.0] | 20.3 [14.6, 22.9] | 0.70 |
| 2h post-exposure | 18.3 [12.0, 23.3] | 13.9 [10.2, 25.8] | 0.78 |
| 24h post-exposure | 14.6 [12.7, 15.4] | 16.2 [9.5, 26.7] | 0.62 |
| IL-4, pg/mL |  |  |  |
| pre-exposure | 36.9 [23.6, 50.2] | 45.1 [33.8, 73.8] | 0.23 |
| 2h post-exposure | 24.6 [14.4, 35.9] | 45.1 [15.4, 98.4] | 0.23 |
| 24h post-exposure | 51.3 [14.4, 86.1] | 49.2 [17.4, 77.9] | 0.88 |
| IL-5, pg/mL |  |  |  |
| pre-exposure | 117.9 [72.5, 142.8] | 126.9 [95.2, 185.9] | 0.27 |
| 2h post-exposure | 95.2 [68.0, 111.1] | 108.8 [72.5, 185.9] | 0.34 |
| 24h post-exposure | 99.7 [81.6, 208.5] | 108.8 [79.3, 185.9] | 0.98 |
| IL-10, pg/mL |  |  |  |
| pre-exposure | 18.7 [8.3, 21.3] | 16.5 [9.4, 29.2] | 0.61 |
| 2h post-exposure | 11.0 [7.2, 14.8] | 13.2 [8.3, 23.1] | 0.41 |
| 24h post-exposure | 17.8 [11.4, 22.3] | 27.5 [17.6, 34.4] | 0.12 |
| IL-12, pg/mL |  |  |  |
| pre-exposure | 9.7 [6.5, 15.3] | 9.9 [9.6, 16.1] | 0.52 |
| 2h post-exposure | 6.8 [4.7, 10.3] | 10.9 [7.1, 17.7] | 0.14 |
| 24h post-exposure | 10.0 [9.2, 30.3] | 9.9 [7.4, 16.0] | 0.64 |
| IL-13, pg/mL |  |  |  |
| pre-exposure | 48.8 [44.7, 69.1] | 48.8 [48.8, 65.0] | 0.65 |
| 2h post-exposure | 65.0 [44.7, 69.1] | 65.0 [65.0, 93.5] | 0.16 |
| 24h post-exposure | 73.2 [32.5, 85.4] | 65.0 [50.8, 65.0] | 0.66 |
| TGF-beta1, pg/mL |  |  |  |
| pre-exposure | 4368.1 [698.6, 6960.4] | 4019.1 [2555.6, 8051.4] | 0.70 |
| 2h post-exposure | 4204.1 [120.8, 12952.2] | 3368.9 [2038.9, 3770.6] | 0.64 |
| 24h post-exposure | 3621.7 [1570.0, 11273.7] | 4257.9 [3084.7, 8161.2] | 0.99 |
| TNF-alpha, pg/mL |  |  |  |
| pre-exposure | 45.1 [41.1, 53.9] | 45.1 [29.4, 72.5] | 0.96 |
| 2h post-exposure | 43.1 [17.6, 63.7] | 43.1 [35.3, 75.2] | 0.24 |
| 24h post-exposure | 41.1 [34.3, 85.2] | 45.1 [29.4, 74.4] | 0.68 |
